# The Natural History of the Development and Resolution of Achilles and Patellar Tendon Sonographic Abnormalities in a Collegiate Cohort

**DOI:** 10.1155/tsm2/1458964

**Published:** 2025-08-05

**Authors:** Luke A. Johnson, Kristin S. Hilger, Shelby Mills, Derek Stokes, Ellen Casey, Sarah F. Eby, Daniel M. Cushman

**Affiliations:** ^1^Department of Physical Medicine and Rehabilitation, University of Utah, Salt Lake City, Utah, USA; ^2^Department of Orthopaedics, University of Utah, Salt Lake City, Utah, USA; ^3^School of Medicine, University of Utah, Salt Lake City, Utah, USA; ^4^College of Osteopathic Medicine of the Pacific Northwest, Western University of Health Sciences, Lebanon, Oregon, USA; ^5^Department of Physiatry, Hospital for Special Surgery, New York, New York, USA; ^6^Department of Physical Medicine and Rehabilitation, Spaulding Rehabilitation Hospital, Harvard Medical School, Boston, Massachusetts, USA

## Abstract

**Purpose:** To prospectively identify the development and regression of Achilles and patellar sonographic abnormalities in collegiate athletes.

**Methods:** Prior to the beginning of their seasons, the Achilles and patellar tendons of collegiate athletes were sonographically videoed by an experienced sonographer. Subjects were then re-recorded at the end of 1 year of competition in an identical manner. Measurements were obtained using consistent predetermined protocols for each participant. Videos of the results were assessed in a blinded manner for echogenicity, tendon thickening, and neovascularization.

**Results:** A total of 147 patellar and 148 Achilles tendons were recorded, with 40.1% of patellar and 16.2% of Achilles tendons identified to have abnormalities at baseline. Of all tendons analyzed, zero patellar and one Achilles tendon were transformed from “abnormal” to “normal”—this single tendon showed only a single neovessel without hypoechogenicity or thickening. Of all tendons initially categorized as “normal,” only 4 patellar tendons switched categories to “abnormal” by the second scan, all with new hypoechogenic foci. Amongst these, all participants were asymptomatic.

**Conclusions:** This prospective study demonstrated that all Achilles and patellar tendons with sonographic abnormalities remain abnormal after 1 year of training and competition, with the exception of a single neovessel on one Achilles tendon that disappeared. A small percentage of collegiate student-athletes developed new abnormalities over a year of practice and competition. This may refute the idea that tendinosis comes and goes in this athletic population, given the minimal change in categorization of participants from either category.

## 1. Introduction

Achilles and patellar tendinopathy are common injuries in the athletic and general populations, requiring significant time away from sport and activities [1–3]. Ultrasound is a reliable modality for detecting the structural abnormalities of Achilles and patellar tendinopathy [4, 5], typically demonstrating one or more of the following: hypoechogenic change, tendon thickening, and/or neovascularization [6]. In general, these abnormalities can develop from overuse, metabolic changes, genetics, disease, injury, or inflammation [7, 8]. Though sonographic abnormalities are commonly observed in painful tendons, tendon abnormalities can also be seen in asymptomatic people [6, 9–11]. The exact relationship between these abnormalities and future injuries or pain is still debated, with mixed results in the literature [6, 12].

The natural history and temporal nature of the development (and possibly disappearance) of tendon abnormalities are poorly understood. It is known that tendon adaptation occurs with repeated tendon loading such as running, and differences in sonographic appearance can be seen [13–15]. In addition, the question of which athletes develop asymptomatic tendon abnormalities, or which athletes revert back to normal tendon morphology from previously abnormal morphology, has not been addressed within the last 2 decades. Prior studies have shown some appearance and disappearance of sonographic abnormalities [16–19], but most were performed at a time when sonographic spatial resolution was inferior to today's abilities.

This study aimed to prospectively identify the natural history of the development and regression of Achilles and patellar sonographic abnormalities in a collegiate athletic population and to identify risk factors for these changes.

## 2. Materials and Methods

This research was conducted as a subanalysis from a larger, multi-institutional study that included three Division I NCAA institutions and was approved by the primary author's institutional review board. For the larger study, student-athletes were asked to participate during their pre-season physical examinations during the summers of 2021 and 2022. With consent, participants responded to a survey to obtain demographic information and then had bilateral patellar and Achilles tendons sonographically examined and recorded. Exclusion criteria were as follows: (1) age under 18 or (2) surgery/amputation to the tendon of interest performed between the two examinations (including ACL surgery with patellar tendon grafts). Any tendons that had previously undergone surgery were excluded as well.

### 2.1. Ultrasound Examinations

A 12–18 MHz linear probe (Logiq E9 R7, Sonosite PX, Sonosite X-Porte, or Konica Minolta Sonimage HS-1) was used for the ultrasound scans. All ultrasound scans were performed using the same predetermined protocol: right patellar tendon, left patellar tendon, right Achilles tendon, and left Achilles tendon. Sports medicine physicians with at least 5 years of post-training experience supervised or performed the scans. Student-athletes were initially placed in the supine position with their knees bent at 90° for long- and short-axis patellar tendon imaging. Next, their legs were fully extended and relaxed for patellar neovascularization detection and recording. Neovascularity was measured and recorded using a previously validated method [20], using power Doppler with minimal probe pressure and increased color gain. Participants were then transitioned to a prone position with their ankles passively flexed to 0° dorsiflexion for Achilles tendon long- and short-axis imaging. The ankles were then relaxed to their natural resting position for Achilles neovascularization measurements. All tendon ultrasound data were stored as videos, lasting approximately 10 s for each view: 3 videos per tendon (long-axis, short-axis, and color power Doppler), or a total of 12 videos per subject.

### 2.2. Video Analysis

For the subanalysis, video assessment of the patellar and Achilles tendons was performed at a later time by two investigators on all athletes who had ultrasound scans at both timepoints. Both reviewers were blinded to any identifying information or athlete characteristics for the tendons under review. Any athlete who had a scan at both timepoints was included. Any interpretation discrepancies were decided by the senior investigator. Assessments included identifying the presence and measurement of hypoechoic focal areas within the tendons, morphologic tendon thickening, and neovascularity. Echogenicity was categorized as normal or abnormal (hypoechoic). Thickness was described as either normal or abnormal (morphologic convexity). Neovascularity was categorized as absent or present. A pilot sample of 40 athletes (80 tendons) demonstrated a near-to-perfect inter-rater reliability in identification of abnormalities between two experienced sonographers (kappa = 0.947 for patellar tendon and 1.000 for Achilles tendon).

## 3. Results

One subject underwent ACL reconstruction with a patellar tendon graft during the study period, so that particular patellar tendon was excluded from the analysis; otherwise, there were 147 patellar tendons and 148 Achilles tendons included in 74 subjects (Table 1). Baseline testing revealed abnormalities in 59 (40.1%) patellar and 24 (16.2%) Achilles tendons.

**Figure 1:**
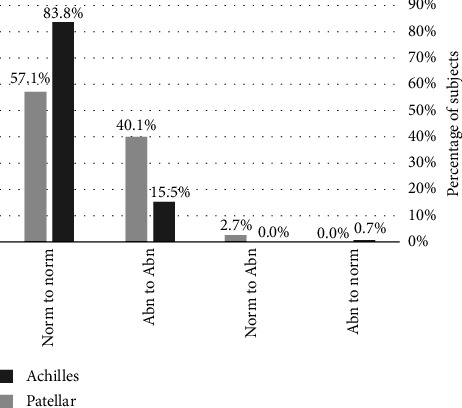
Sonographic changes seen over the study period for all subjects (*n* = 148 Achilles tendons, *n* = 147 patellar tendons). Norm = sonographically normal tendon, Abn = tendon with at least one sonographic abnormality.

**Figure 2:**
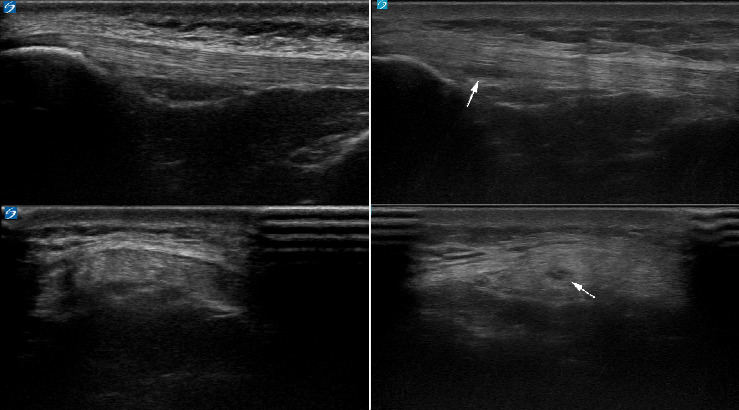
22-year-old male lacrosse athlete who developed an area of hypoechogenicity in the inferior aspect of the proximal patellar tendon. Left images are the tendon at initial timepoint, right images are the tendon 1 year later.

**Figure 3:**
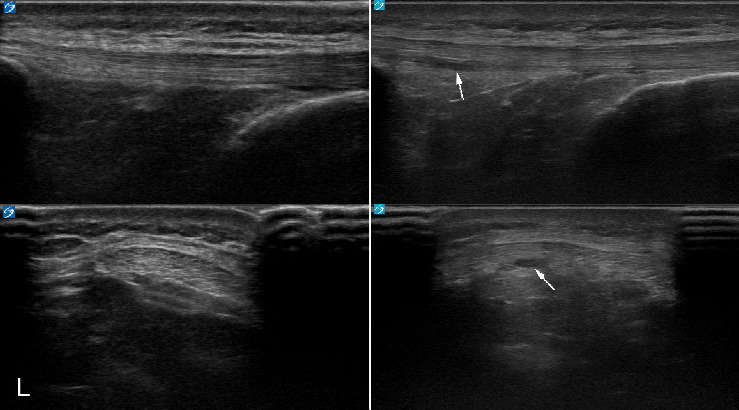
21-year-old male lacrosse athlete who developed areas of hypoechogenicity in the deep aspect of the left patellar tendon. Left images are the tendon at initial timepoint, and right images are the tendon 1 year later.

**Figure 4:**
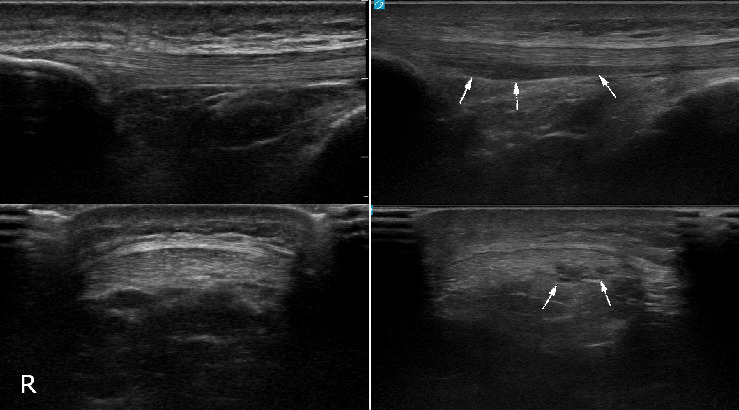
21-year-old male lacrosse athlete who developed areas of hypoechogenicity in the deep aspect of the right patellar tendon. Left images are the tendon at initial timepoint, right images are the tendon 1 year later.

**Figure 5:**
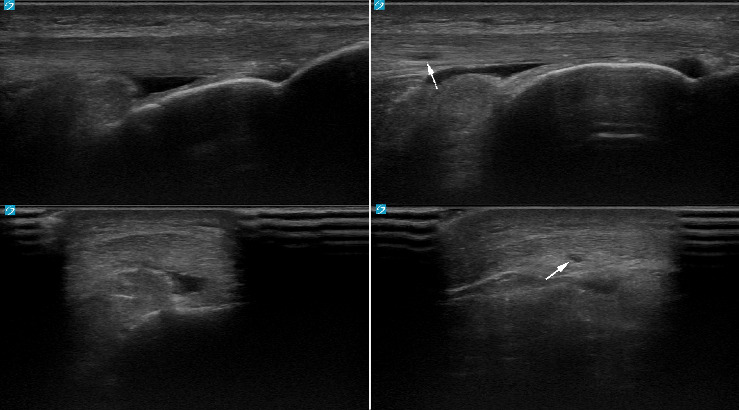
24-year-old male basketball athlete who developed an area of hypoechogenicity in the deep aspect of the right patellar tendon. Left images are the tendon at initial timepoint, right images are the tendon 1 year later.

**Figure 6:**
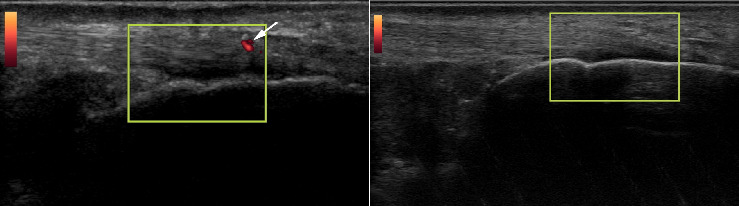
20-year-old female beach volleyball athlete who initially had a single neovessel at the insertional aspect of the superficial right Achilles tendon. Left image is the tendon at initial timepoint, right image is the tendon 1 year later.

Figure 1 depicts the patellar and Achilles tendon morphological categorizations from year one to year two for the study subjects. Notably, no patellar tendons categorized as abnormal at the initial timepoint were characterized as normal on follow-up testing, while one (0.7%) abnormal Achilles tendon on initial evaluation appeared sonographically normal on follow-up testing. No Achilles tendons categorized as normal at the initial timepoint developed sonographic abnormalities over the study period, while 4 (2.7%) patellar tendons did. Figures 2, 3, 4, and 5 demonstrate the evolution of sonographic findings in all subjects that experienced sonographic tendon changes during the study. The largest novel (patellar) abnormality over the study period measured 2 × 5 × 20 mm. The single subject whose tendon changed from abnormal to normal (Figure 6) had a single neovessel at the initial timepoint, but no increased thickness or hypoechogenicity at either timepoint. All subjects who developed new abnormalities over the year were pain-free at the time of the second scan.

## 4. Discussion

The results of this study demonstrate that a majority of collegiate athletes have normal sonographic tendon morphology at baseline and remain so after 1 year of training and competition. Importantly, the converse is also true; once tendon morphology appears abnormal (the presence of focal hypoechogenicity, morphologic thickening, and/or neovascularity), it remains abnormal a year later. The single example of a tendon changing from abnormal to normal was characterized by no morphologic abnormalities, but only the presence of a single neovessel, which is not necessarily a sign of abnormal morphology [21–23]. Furthermore, only a small percentage (3%) of subjects' tendons demonstrated changes from normal to abnormal in this collegiate cohort.

### 4.1. Tendons That Changed From Abnormal Back to Normal

Of the 24 Achilles tendons and 59 patellar tendons initially categorized as abnormal, none returned to normal on sonography after one year of examination, with the exception of a single neovessel disappearing in the Achilles tendon. It is well documented that tendons injured in fetal development can repair themselves, returning to normal measures and morphology [24, 25]. Reports of injured mature tendons returning to normal morphology are mixed, however, with older literature [16–19] suggesting tendons can return to normal in adults but more recent reviews suggesting they do not [26]. This may in large part be related to sonographic resolution, which has improved significantly over the last couple of decades, as small abnormalities may not have been able to be seen as easily. A more recent study of patellar tendons matched the present findings, where no tendons reverted to normal once they were abnormal [27]. Furthermore, it is unclear from the early studies exactly how they were evaluated. In this study, video capture allowed a complete view of the tendon at both timepoints for full comparison at a later timepoint. These results suggest that once mature Achilles or patellar tendons develop an abnormality, they remain abnormal. This is consistent with more recent literature suggesting that the stage of tendon injury, age, and tendon location are factors that determine if the structure can return to normal morphology or not [28].

In the single Achilles tendon that changed from abnormal to normal, the only abnormality noted was neovascularization. Neovascularity has been shown to decrease over time in competitive athletes [29]. However, neovascularization alone (i.e., not in the presence of hypoechogenicity and/or morphologic thickening) does not necessarily indicate tendinopathic change. Neovascularization without other signs of tendinopathic change could also be seen in the setting of inflammatory enthesopathy such as spondyloarthropathies (e.g., psoriatic arthritis), infection, or other problems not directly related to tendon overload [30], or it may be related to exercise [31].

### 4.2. Tendons That Changed From Normal to Abnormal

Of the 88 patellar and 124 Achilles tendons initially categorized as normal, only 2.7% of the patellar and none of the Achilles tendons changed to abnormal over the course of a year of competitive athletic activity. The prior literature, from 2 decades ago, demonstrated a mixed amount of progression of sonographic abnormalities over time, both in subjects slightly older than the present study. Fredberg and Bolvig [19] showed that only 1.2% of Achilles and 7.5% of patellar tendons changed from normal to abnormal over a year in professional football (soccer) athletes. Cook et al. [18] identified that 25% of patellar tendons changed from normal to abnormal over a longer time period (4 years). The findings in the present study may refute the idea that tendinosis comes and goes in this population, given the minimal change in categorization of participants from either category. A more recent study identified that patellar tendon in adolescent basketball players had 9% of their patellar tendons change from normal to abnormal over 2.5 years [27]. The present study did not assess the degree of sonographic abnormality (e.g., the size of the abnormality, the degree of neovascularity, the location of the abnormality, etc.) in subjects with pre-existing abnormalities, so there may have been an increase or decrease in their size/morphology.

Further, imaging abnormalities alone do not indicate whether the tendon will be symptomatic at the time of scanning, or in the future. In fact, all subjects whose tendons changed from normal to abnormal remained asymptomatic in this cohort. The positive predictive value of a sonographic abnormality progressing to symptomatic tendinopathy remains quite low [6]. Many patients have sonographically abnormal tendon appearance without pain [12]. Abnormal sonographic findings have been identified more consistently in athletes than in the nonathlete population, even without symptoms [32, 33]. This study therefore only demonstrates that abnormalities in this population tend to stay abnormal, and are not necessarily reflective of symptoms. Study comparisons have not been performed between adult competitive athletes and immature athletes or elite athletes, though it is realistic that healing properties may differ in these populations.

### 4.3. Limitations of This Study

This study was limited in the frequency and duration of monitoring, with only two scans total over the course of 1 year. As such, there exists a possibility for alteration between normal and abnormal and vice versa in the interim, which was not observed by our discrete imaging timepoints. A longer duration for ultrasound imaging follow-up could also support results seen by previous investigators, which found larger percentages of subjects becoming abnormal over a longer period of time [18]. Importantly, this study does not examine symptomatic tendinopathy, only the sonographic appearance of the tendon.

### 4.4. Strengths of This Study

Each sonogram was recorded by sports medicine physicians with at least 5 years of post-training experience in the field, using modern devices with high-quality spatial resolution. By using ultrasound video collection, more comprehensive analysis and interpretation could be performed with very high inter-rater reliability, unlike previous studies which used still images [19, 27, 34]. The delayed and blinded image analysis also decreased risk of bias.

## 5. Conclusions

This study demonstrated that in collegiate athletes, a majority of patellar and Achilles tendons were normal-appearing on ultrasound. Those that were abnormal with focal hypoechogenic areas and/or morphologic thickening remained abnormal over the course of a calendar year with regular training and competition. Few tendons, all patellar, transitioned from normal to abnormal. This study suggests that sonographically abnormal tendons in this population stay abnormal over a 1-year time period. Additional studies, with increased longitudinal follow-up of tendon abnormalities over time in patients of varying ages will increase our understanding of the current findings and tendon repair and morphology. Continued research on the relationship between tendon abnormalities and their association to dysfunction, tendon development and repair, and tendon therapies is warranted.

## Figures and Tables

**Table 1:** Tendon counts by type.

Tendon type	Total tendons included	Notes
Patellar tendon	147	One tendon excluded due to ACL reconstruction with patellar graft
Achilles tendon	148	No exclusions
Subjects	74	

## Data Availability

The data that support the findings of this study are available on request from the corresponding author. The data are not publicly available due to privacy or ethical restrictions.
